# Prognostic Impact of Combined Nutritional and Cognitive Status on Long-Term Outcome in Acute Decompensated Heart Failure

**DOI:** 10.3390/nu18020189

**Published:** 2026-01-06

**Authors:** Kazutaka Nogi, Tomoya Ueda, Atsushi Kyodo, Satomi Ishihara, Yasuki Nakada, Yukihiro Hashimoto, Hitoshi Nakagawa, Taku Nishida, Ayako Seno, Kenji Onoue, Makoto Watanabe, Yoshihiko Saito, Shungo Hikoso

**Affiliations:** 1 Department of Cardiovascular Medicine, Nara Medical University, Kashihara 634-8522, Japan; nogi18kazu@dream.jp (K.N.);; 2 Department of Cardiovascular Medicine, Nara Prefecture Seiwa Medical Center, Sango 636-0802, Japan

**Keywords:** geriatric nutritional risk index, heart failure, mini-mental state examination, elderly

## Abstract

**Background/Objectives:** Malnutrition and cognitive impairment are both common and prognostically significant in elderly patients with acute decompensated heart failure (ADHF). However, the combined impact of nutritional and cognitive status on long-term outcomes remains unclear. This study aimed to evaluate the prognostic value of the Geriatric Nutritional Risk Index (GNRI) and Mini-Mental State Examination (MMSE) in elderly patients hospitalized for ADHF. **Methods:** We analyzed 414 ADHF patients aged ≥65 years from the NARA-LONGEVITY study. Patients were categorized into four groups based on GNRI (≥92 or <92) and MMSE (>23 or ≤23) values at discharge. The primary endpoint was a composite of all-cause mortality and HF-related readmission. **Results:** During a median follow-up of 37.4 months, 218 patients (52.7%) reached the composite endpoint, and 168 (40.6%) died. Patients with both low GNRI and low MMSE had significantly poorer outcomes than those with high GNRI and high MMSE (adjusted hazard ratio [HR] for composite outcome: 2.16; 95% CI, 1.28–3.64; *p* = 0.004; HR for all-cause mortality: 2.21; 95% CI, 1.22–3.99; *p* = 0.009). The combined prognostic impact was consistent across age subgroups. **Conclusions:** The combined assessment of nutritional and cognitive status using GNRI and MMSE at discharge provides additional prognostic value in elderly patients with ADHF. These findings highlight the importance of a multidimensional approach to risk stratification and personalized care planning in this population.

## 1. Introduction

Heart failure (HF) is a significant global health challenge, with its prevalence steadily increasing in parallel with population aging [1]. Elderly individuals constitute the fastest-growing segment of patients hospitalized for HF, and account for a substantial proportion of admissions for acute decompensated heart failure (ADHF) [2]. Epidemiological data demonstrate that older patients with ADHF not only experience higher rates of rehospitalization and long-term mortality than younger patients [3], but also face unique management challenges owing to multimorbidity and functional decline [4]. Despite advances in pharmacological and device-based therapies [5,6,7], outcomes in this population remain suboptimal, highlighting the urgent need for reliable and practical tools to improve risk stratification [8]. Among the diverse comorbidities associated with poor prognosis in elderly HF patients, malnutrition and cognitive impairment are particularly prevalent and clinically important.

Malnutrition is prevalent among elderly HF patients and linked to systemic inflammation, sarcopenia, immune dysfunction, and reduced physiological reserve [9,10], thereby accelerating HF progression [11]. Nutritional risk in HF can be assessed using questionnaire-based tools such as the Mini Nutritional Assessment (MNA) and Subjective Global Assessment (SGA), as well as laboratory-based indices including the Controlling Nutritional Status (CONUT) score, Prognostic Nutritional Index (PNI), and Geriatric Nutritional Risk Index (GNRI) [12]. Systematic reviews and meta-analyses consistently demonstrate that malnutrition assessed by these tools is associated with increased mortality and HF-related rehospitalization [12]. Among these many indicators, GNRI, a composite measure based on serum albumin (Alb), height, and body weight (BW), was originally developed for geriatric populations and allows objective nutritional risk assessment without requiring patient cooperation [13]. This feature is particularly important in elderly patients hospitalized for ADHF, in whom cognitive impairment, delirium, and severe symptoms frequently limit the feasibility of subjective assessments. Moreover, GNRI has since been validated as a simple and reliable tool for assessing nutritional risk in heart failure and has been consistently shown to be strongly associated with increased mortality, higher rates of rehospitalization, and adverse cardiovascular events [9,14,15,16,17,18].

Cognitive impairment is also highly prevalent among elderly patients with HF, affecting up to 40–60% in some cohorts [19,20,21]. Even mild impairment has been associated with increased long-term mortality, reduced quality of life, and greater risk of readmission [22]. The Mini-Mental State Examination (MMSE) is a widely used screening tool designed to assess cognitive function in clinical practice [23]. Prior studies have consistently shown that lower MMSE scores correlate with worse outcomes, partly due to impaired self-care, medication non-adherence, and difficulties in engaging with complex treatment regimens [19,22,24].

Although both GNRI and MMSE have individually been recognized as important prognostic markers in HF, they are typically examined in isolation. Each represents distinct but potentially complementary dimensions of patient vulnerability: GNRI reflects biological and metabolic reserve [14], while MMSE reflects neurocognitive and behavioral capacity [24]. Importantly, malnutrition and cognitive decline may also interact bidirectionally: inadequate nutrition can exacerbate frailty and brain dysfunction [25], whereas cognitive impairment may worsen nutritional intake and treatment adherence [26,27].

However, the combined prognostic impact of nutritional and cognitive status in elderly patients with ADHF has not been systematically evaluated. Prior studies have not determined whether integrating GNRI and MMSE provides incremental prognostic information beyond either measure alone [22,28]. To address this gap, we hypothesized that a combined assessment could yield synergistic and clinically actionable insights, enabling more robust and meaningful post-discharge risk stratification than single-domain evaluation.

## 2. Materials and Methods

### 2.1. Study Design and Population

The NARA-LONGEVITY study is a prospective cohort study that enrolled 1745 consecutive patients admitted to the emergency department or coronary care unit of our hospital with documented ADHF (either acute new-onset or acute-on-chronic HF) or acute myocardial infarction (AMI) between 1 January 2016, and 31 December 2022. Of these, 827 patients were hospitalized for ADHF. The diagnosis of HF was based on the Framingham Criteria [29]. For the present analysis, we applied the following criteria:

Inclusion criteria: age ≥ 65 years and hospitalization for ADHF.

Exclusion criteria: AMI, in-hospital death, or missing MMSE data.

ADHF cases triggered by AMI were excluded to maintain a clinically homogeneous ADHF population, as AMI-related HF represents a distinct phenotype with differing pathophysiology, inflammatory and catabolic responses, and potential differences in nutritional dynamics and cognitive trajectories, which could confound interpretation of discharge-time GNRI–MMSE prognosis. Among the 271 patients without MMSE data, 49 died during the index hospitalization and were therefore ineligible for cognitive testing; the remaining 222 discharge survivors without MMSE were excluded from the primary analytic cohort but are described in Supplementary Table S2 for comparison with the 414 patients who underwent MMSE assessment.

After exclusions, 414 patients were included in the present analysis, and were categorized into four groups according to the high/low-GNRI (≥92, <92) and high/low-MMSE (>23, ≤23) values at discharge: high-GNRI/high-MMSE, low-GNRI/high-MMSE, high-GNRI/low-MMSE, and low-GNRI/low-MMSE groups. We investigated the impact of the combined assessment of GNRI and MMSE scores on the prognosis of ADHF. The Ethics Committee of Nara Medical University approved the study protocol (approval number: 1035). Written informed consent was obtained from all patients in accordance with the Declaration of Helsinki’s Ethical Principles for Medical Research Involving Human Subjects.

### 2.2. Data Collection and Definitions

Baseline demographics, comorbidities, vital signs, and laboratory measurements [hemoglobin (Hb), Alb, blood urea nitrogen (BUN), creatinine, estimated glomerular filtration rate (eGFR) calculated using the diet modifications of the renal disease method, serum electrolytes (sodium and potassium), and B-type natriuretic peptide (BNP)] were obtained at discharge. Echocardiography was performed in all patients at discharge. Medication records included angiotensin-converting enzyme inhibitor (ACE-I), angiotensin II receptor blocker (ARB), angiotensin receptor neprilysin inhibitor (ARNI), beta-blockers, aldosterone antagonists, and sodium–glucose cotransporter 2 (SGLT2) inhibitors. For loop diuretics other than furosemide, doses were converted to furosemide equivalent doses: 4 mg of torasemide and 30 mg of azosemide were each considered equivalent to 20 mg of furosemide [30,31]. In routine discharge decision-making, objective markers of congestion—such as inferior vena cava (IVC) diameter, BNP trends, and clinical or diuretic response—were reviewed to confirm clinical stabilization and guide volume management.

### 2.3. Assessment Tools

#### 2.3.1. Geriatric Nutritional Risk Index

GNRI was calculated using serum Alb, height, and BW [13]. Serum Alb and BW were measured at discharge after clinical optimization. Based on this study, we divided patients into two nutrition related risk groups as follows: the low-GNRI group (GNRI < 92) and a high-GNRI group (GNRI ≥ 92) in our study (The detailed calculation formula and cut-off derivation are provided in Supplementary Table S1).

#### 2.3.2. Mini-Mental Status Examination

Cognitive function was assessed at discharge using the MMSE, which evaluates multiple domains (orientation, registration, attention, recall, language, and visuospatial ability) on a 30-point scale [32]. We divided patients into two cognitive function related risk groups as follows: a low-MMSE group (MMSE ≤ 23) and a high-MMSE group (MMSE > 23) in our study (Domain structure and scoring details are provided in Supplementary Table S1). The MMSE was administered face-to-face after clinical stabilization as part of routine transitional care by certified cardiology resident under the supervision of attending cardiologists. Although formal inter-rater reliability testing was not embedded in the original registry design, all assessors received standardized MMSE training before participation, including structured lectures, demonstration cases, supervised real-patient practice, and periodic feedback on scoring procedures. MMSE was typically not performed when patients exhibited delirium, hemodynamic instability, impaired consciousness, severe exhaustion, or inability to communicate or cooperate, conditions that frequently preclude cognitive testing in real-world acute HF care.

### 2.4. Outcomes

The primary endpoint was a composite of all-cause death and HF readmission in the time-to-event analysis. The secondary endpoint was postdischarge all-cause death in time-to-event analysis. Patient statuses were assessed using medical records and information from participating cardiologists. When these data were unavailable, the clinicians sent letters to the patients’ homes or telephoned them or their families to request the data.

### 2.5. Statistical Analysis

Continuous variables were expressed as mean ± standard deviation for normally distributed data and as median with interquartile range for non-normally distributed data. Categorical variables were expressed as counts and percentages. Differences between groups were compared using the ANOVA or Kruskal–Wallis test for continuous variables and the chi-square test for categorical variables.

To minimize bias due to missing data and avoid loss of power, we used multiple imputations by chained equations (MICE), generating 20 imputed datasets under a missing-at-random assumption. Continuous variables were imputed using predictive mean matching, categorical variables using classification and regression trees (CART), and ordinal variables (e.g., NYHA class) using proportional odds logistic regression. GNRI values were recalculated after imputation from serum albumin, height, and body weight. MMSE scores were imputed as continuous variables and subsequently rounded and clipped to remain within the valid range (0–30). The imputation models included all baseline covariates and outcome indicators, but survival time and event status were not imputed. Patients without any MMSE assessment (i.e., completely missing MMSE, *n* = 271 including 49 in-hospital deaths) could not be classified into GNRI–MMSE strata and were excluded a priori from the primary analytic cohort; multiple imputation was used only for sporadically missing covariates. As a sensitivity analysis, we also performed a complete-case Cox analysis excluding patients with any missing covariate data.

Time-to-event outcomes were analyzed using Kaplan–Meier curves with log-rank tests. Cox proportional hazards regression models were constructed to estimate hazard ratios (HRs) and 95% confidence intervals (CIs) for each group, using the High GNRI–High MMSE group as the reference. Multivariable Cox models adjusted for clinically relevant covariates known to influence HF prognosis: age, sex, New York Heart Association (NYHA) functional classification, diabetes mellitus, Hb, BNP, left ventricular ejection fraction (LVEF) at discharge, BUN, creatinine, serum sodium, systolic blood pressure at discharge, and discharge use of renin–angiotensin system inhibitors (ACE inhibitors, ARBs, or ARNIs), beta-blockers, aldosterone antagonists, and sodium glucose cotransporter 2 (SGLT2) inhibitors [33]. The proportional hazards assumption was verified using Schoenfeld residuals.

Because nutritional status and cognitive function are conceptually related, we examined their interrelationship and potential collinearity. The association between GNRI and MMSE at discharge was assessed using Spearman’s rank correlation coefficient. Multicollinearity in the Cox models was evaluated using variance inflation factors (VIFs) for GNRI, MMSE, and all covariates.

Given the strong influence of age on both nutritional status and cognition, all multivariable models were adjusted for age. In addition, we performed age-acknowledged sensitivity analyses: (i) a Cox model including age as a continuous covariate (per year), and (ii) a model in which age was modeled flexibly using natural cubic splines. In the spline-based model, we formally tested for an age × GNRI–MMSE group interaction using a likelihood-ratio test to assess whether the prognostic impact of the four GNRI–MMSE categories varied across the age spectrum. To further illustrate the effect of age, we also estimated age-standardized absolute risks of the composite endpoint across GNRI–MMSE groups using g-computation based on the multivariable Cox model.

To address potential information loss from dichotomizing GNRI and MMSE, we also fitted multivariable Cox models treating GNRI and MMSE as continuous variables. In these analyses, GNRI and MMSE were entered as standardized variables (per 1-SD increase) and adjusted for the same covariates as in the primary model, including age. To explore departures from linearity, we further modeled GNRI and MMSE using restricted cubic splines with three degrees of freedom in separate Cox models, again adjusting for the complete covariate set. Predicted hazard ratio curves with 95% CIs from these spline models are presented in the Supplementary Figures.

Because approximately one-third of eligible ADHF patients were excluded from the primary cohort owing to missing MMSE scores, we performed prespecified sensitivity analyses to evaluate the potential impact of this exclusion. First, we used inverse probability weighting (IPW) based on the estimated probability of undergoing MMSE assessment, derived from a logistic regression model including baseline demographics, comorbidities, vital signs, laboratory data, and discharge medications corresponding to the main Cox model. Second, we constructed a missing-indicator Cox model including all discharge survivors (both with and without MMSE) and adding a binary indicator for missing MMSE. In both approaches, the GNRI–MMSE risk gradient was re-evaluated, and the independent prognostic contribution of a “missing MMSE” indicator was examined. Baseline characteristics of patients with and without MMSE assessment are summarized in Supplementary Table S2.

Parameter estimates from Cox models fitted in each imputed dataset were combined using Rubin’s rules. Statistical significance was defined as a two-sided *p* value < 0.05. All statistical analyses were performed using R software version 4.1.2 (R Foundation for Statistical Computing, Vienna, Austria). Because this was a secondary analysis of a pre-existing prospective cohort, no formal a priori sample size calculation was performed. However, a post hoc calculation indicated that with 414 patients and an observed composite event rate of 52.7%, the study had >80% power to detect a hazard ratio of 1.6 or greater between the lowest- and highest-risk groups at a one-sided α = 0.05.

## 3. Results

### 3.1. Patient Characteristics

**Figure 1:**
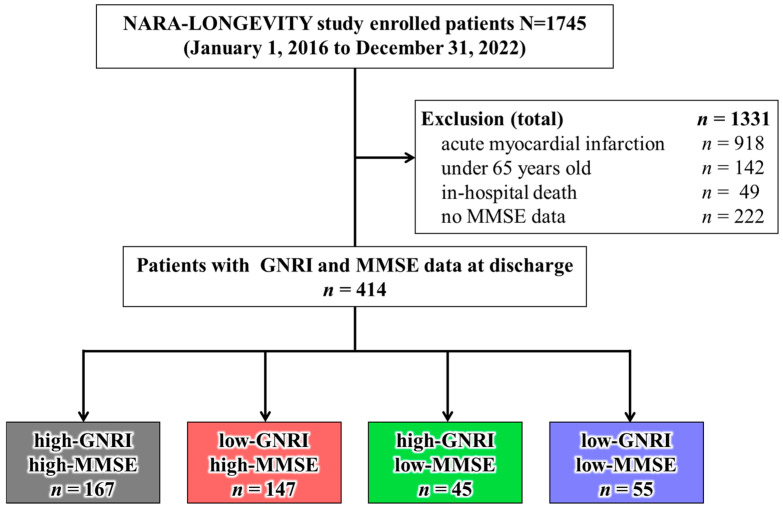
Flow chart of the study cohort.

Figure 1 shows the enrolment criteria, exclusion criteria, and study flow. Of the patients enrolled in the NARA-LONGEVITY study, 414 were included in the present study, excluding those with AMI (*n* = 918), those under 65 years of age (*n* = 142), in-hospital death (*n* = 49), and those without MMSE data (*n* = 222). Among them, the high-GNRI/high-MMSE, low-GNRI/high-MMSE, high-GNRI/low-MMSE, and low-GNRI/low-MMSE groups comprised 167 (40.3%), 147 (35.5%), 45 (10.9%), and 55 (13.3%) patients, respectively. Baseline characteristics of discharge survivors with and without MMSE assessment are compared in Supplementary Table S2. Patients without MMSE tended to be older, more comorbid, and less likely to receive guideline-directed medical therapies than those included in the analytic cohort, suggesting a higher frailty and illness-severity burden among the MMSE-missing subgroup.

NARA-LONGEVITY study enrolled a total of 827 patients. We excluded a total of 413 patients (under 65 years old and no MMSE data). We divided 414 patients into four groups according to the high and low GNRI and MMSE values at discharge.

**Figure 2:**
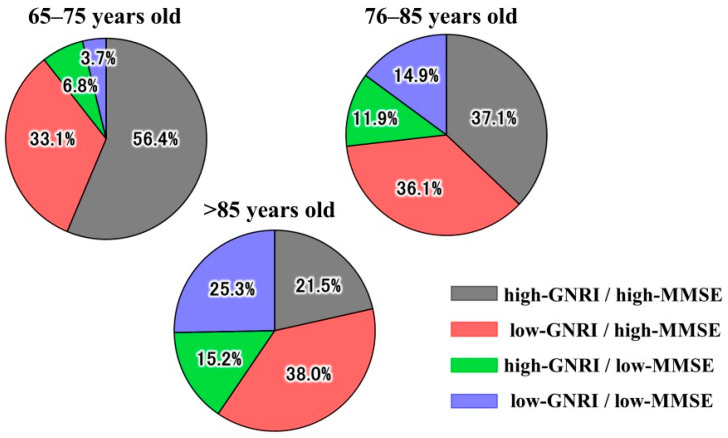
Prevalence of combined groups of GNRI and MMSE at discharge in the total cohort and according to age category.

The median age of the patients was 79 (range, 73–84) years, and 57.5% were men. The prevalence of high-GNRI/high-MMSE decreased and the prevalence of low-GNRI/low-MMSE increased with age (*p* for trend < 0.001, and 0.001). More than three-quarters of elderly patients aged >85 years had low GNRI or low-MMSE (Figure 2). Baseline patient characteristics stratified by high/low GNRI and MMSE scores are shown in Table 1. Age was significantly higher in the low-GNRI/low-MMSE group than in the other groups. BMI was significantly higher in the high-GNRI groups than in the low-GNRI groups. There were no significant differences in sex, systolic blood pressure, or heart rate at discharge among the four groups. The proportion of patients with dyslipidemia in the low-GNRI/low-MMSE group was significantly lower than that in the high-GNRI/high-MMSE group. Regarding laboratory parameters, Hb, Alb, and serum sodium levels in the low-GNRI/low-MMSE group were significantly lower than those in the high-GNRI/high-MMSE group. These differences suggest greater metabolic frailty and impaired physiological reserve in patients with combined malnutrition and cognitive impairment. Interestingly, the low-GNRI/high-MMSE group tended to be younger but also displayed markedly reduced BMI and serum sodium, indicating that malnutrition can exist even in relatively cognitively preserved individuals, potentially reflecting an early stage of systemic vulnerability. No significant differences in BNP levels were observed at discharge. LVEF data were available for 410 patients (99.0%), including 138 (33.7%), 103 (25.1%), and 169 (41.2%) with reduced, mid-range, and preserved ejection fractions, respectively. No significant differences in the HF phenotypes were found at discharge.

GNRI and MMSE at discharge were only weakly correlated (Spearman ρ = 0.14, *p* = 0.003), indicating that nutritional status and global cognition captured related but largely distinct dimensions of vulnerability. In the multivariable Cox models, VIFs for GNRI, MMSE, and all adjustment covariates were <1.6, confirming the absence of meaningful multicollinearity.

The prevalence of high-GNRI/high-MMSE decreased and the prevalence of low-GNRI/low-MMSE increased with age (*p* for trend <0.001, and 0.001). More than three-quarters of elderly patients aged >85 years had low GNRI or low-MMSE.

### 3.2. Clinical Outcomes

**Figure 3:**
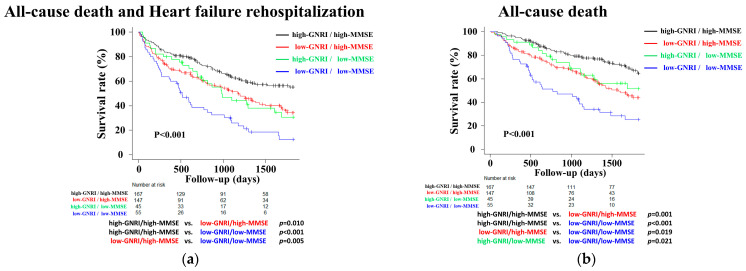
Kaplan–Meier analyses in the combined assessment of the GNRI and MMSE at discharge for postdischarge all-cause death and heart failure rehospitalization (**a**), and all-cause death (**b**).

During the median follow-up period of 37.4 months, 218 composite endpoint events (52.7%) and 168 all-cause deaths (40.6%) were observed. The median time to the composite endpoint was shortest in the low-GNRI/low-MMSE group (20.7 months) compared to the high-GNRI/high-MMSE group (58.9 months). Kaplan–Meier curve analysis showed that the low-GNRI/low-MMSE group had higher event rates for both the composite endpoint and all-cause death than the high-GNRI/high-MMSE group, with the curves demonstrating clear early separation—particularly within the first year—that persisted throughout follow-up (Figure 3a,b). Absolute risk differences between the high- and low-risk groups were 20% at 1 year and 37% at 3 years for the composite endpoint; for all-cause mortality, they were 20% and 38%, respectively.

Multivariable Cox regression confirmed that low-GNRI/low-MMSE status was independently associated with increased risk of adverse outcomes (adjusted HR 2.163 [95% CI 1.284–3.643] for the composite endpoint; adjusted HR 2.209 [95% CI 1.222–3.993] for mortality) (Table 2). The intermediate-risk groups (low-GNRI/high-MMSE and high-GNRI/low-MMSE) showed numerically higher event rates than the high-GNRI/high-MMSE reference, with adjusted HRs consistent with a graded risk pattern across the four strata. Model diagnostics showed no evidence of multicollinearity, the proportional hazards assumption was met using Schoenfeld residuals, and model performance was acceptable (*c*-statistic = 0.70, AIC = 2272).

Given the age imbalance across MMSE strata, we further evaluated the role of age. In a fully age-adjusted Cox model using the same covariates as above, the GNRI × MMSE four-group variable remained significantly associated with the composite endpoint, and age itself was independently related to risk, but did not materially attenuate the prognostic gradient across groups. When age was modeled flexibly using natural cubic splines, the age × GNRI–MMSE group interaction was not significant (likelihood-ratio test *p* = 0.289), indicating that the prognostic impact of the combined nutritional–cognitive categories was not materially modified across the age spectrum. After standardizing for the age distribution using g-computation, the estimated risk of the composite endpoint (all-cause death and HF readmission) remained ordered across the four GNRI–MMSE groups: 91.4% in high-GNRI/high-MMSE, 96.5% in low-GNRI/high-MMSE, 95.9% in high-GNRI/low-MMSE, and 98.5% in low-GNRI/low-MMSE.

To address concerns about information loss from dichotomization, we performed additional Cox models treating GNRI and MMSE as continuous, standardized variables. In these age-adjusted, fully multivariable models, higher GNRI was associated with a lower risk of the composite endpoint (per 1-SD increase, HR 0.81; 95% CI 0.71–0.93; *p* = 0.002), whereas MMSE showed an inverse but statistically non-significant association (per 1-SD increase, HR 0.91; 95% CI 0.81–1.02; *p* = 0.10). Restricted cubic spline analyses for GNRI and MMSE (3 degrees of freedom) demonstrated approximately linear inverse relationships across their central ranges, with widening confidence intervals only at the extremes (Supplementary Figure S1A,B). These findings support the robustness of the main results when GNRI and MMSE are modeled as continuous variables.

Because approximately one-third of eligible ADHF discharge survivors were excluded from the primary analytic cohort due to completely missing MMSE scores, we conducted prespecified sensitivity analyses to evaluate the potential impact of this exclusion. In the inverse probability weighting (IPW) analysis including all 636 discharge survivors (*n* = 414 with MMSE and *n* = 222 without MMSE), reweighted hazard ratios across the four GNRI–MMSE groups closely paralleled those of the primary multivariable Cox models, preserving the original prognostic ordering. In the missing-indicator Cox model, which likewise included all discharge survivors and added a binary indicator for missing MMSE, the GNRI–MMSE risk gradient remained stable and fully preserved. Importantly, the missing-MMSE indicator itself was not independently associated with long-term outcomes after multivariable adjustment (HR 1.03; 95% CI 0.78–1.35), suggesting that the exclusion of patients without cognition testing did not materially distort our primary effect estimates.

In addition, we formally assessed whether age or LVEF modified the prognostic associations of the combined GNRI–MMSE assessment. We tested interactions for both (i) age (≥80 vs. <80 years) and (ii) LVEF (≤40% vs. >40%) using fully covariate-adjusted Cox models. No significant interactions were detected for the composite outcome of all-cause death and HF readmission (interaction *p* = 0.707 for age and *p* = 0.546 for LVEF), or for all-cause death alone (interaction *p* = 0.381 for age and *p* = 0.668 for LVEF), indicating that the prognostic impact of GNRI–MMSE grouping was not materially modified across these age or systolic-function thresholds. (Supplementary Tables S3 and S4).

We further confirmed robustness using lesion reclassification-relevant restriction: sensitivity analyses excluding patients with extreme GNRI values (<82) produced consistent results (Supplementary Figure S2A,B). Finally, a complete-case Cox analysis restricted discharge survivors with no missing covariate data yielded HRs of similar direction, magnitude, and statistical interpretation to those from the primary imputed models, reinforcing the stability of our conclusions.

Kaplan–Meier curve analyses showed that the low-GNRI/low-MMSE group was associated with a higher event rate than the other groups for both the combined endpoint and all-cause death.

Univariate and multivariate Cox proportional hazard analyses were performed among the groups, adjusted for age, sex, NYHA functional classification, diabetes mellitus, Hb, BNP, LVEF at discharge, BUN, Cr, serum sodium, SBP at discharge, ACE-I or ARB or ARNI, beta-blockers, aldosterone antagonists, and SGLT2 inhibitors.

ACE-I, angiotensin-converting enzyme inhibitor; ADHF, acute decompensated heart failure; ARB, angiotensin receptor blocker; ARNI, angiotensin receptor neprilysin inhibitors; BNP, B-type natriuretic peptide; BUN, blood urea nitrogen; CI, confidence interval; Cr, creatinine; GNRI, geriatric nutritional risk index; Hb, hemoglobin; HR, hazard ratio; LVEF, left ventricular ejection fraction; MMSE, mini-mental state examination; NYHA, New York Heart Association functional classification; SBP, systolic blood pressure; SGLT2, sodium-glucose cotransporter 2.

## 4. Discussion

The present study demonstrated that the combined prognostic significance of nutritional and cognitive status, assessed using the GNRI and MMSE, in elderly patients hospitalized for ADHF. We found that patients with concomitant malnutrition and cognitive impairment had substantially higher risks of death and HF readmission than those with preserved nutritional and cognitive status. These findings remained consistent across sensitivity analyses and were not modified by age or LVEF, underscoring the robustness and generalizability of the combined GNRI–MMSE assessment.

### 4.1. Integrative Interpretation and Clinical Significance

Rather than representing fully independent domains, malnutrition and cognitive impairment may represent overlapping manifestations of systemic frailty. Lower albumin, hemoglobin, sodium, and BMI values in the low-GNRI groups likely reflect sarcopenia, chronic inflammation, and impaired metabolic reserve, which may attenuate the efficacy of standard HF therapies and increase vulnerability to decompensation. These laboratory derangements are consistent with a phenotype of systemic frailty. Indeed, prior studies have demonstrated that poor nutritional status is strongly associated with cognitive decline and frailty, conceptualized as “cognitive frailty” [34,35]. In HF populations specifically, malnutrition and cognitive impairment co-occur and jointly contribute to increased vulnerability [26]. Meanwhile, reduced MMSE scores indicate potential difficulties with medication adherence, self-monitoring, and timely care-seeking, all of which can accelerate clinical deterioration. The coexistence of these two conditions therefore identifies a subgroup with both biological and behavioral susceptibility—an especially high-risk phenotype among elderly HF patients.

Interestingly, our registry also revealed a clinically important nuance: nearly one-third of discharge survivors did not undergo MMSE assessment. In routine AHF transitional care, delirium, unstable circulation, profound exhaustion, impaired consciousness, or inability to communicate or cooperate are common in advanced frailty and frequently preclude bedside cognitive testing. While these MMSE-missing patients could not contribute to GNRI–MMSE prognostic quartile stratification, which requires the battery for exposure classification, their exclusion raised the possibility of under-representation of vulnerable phenotypes in the analyzed sample.

To explicitly address these interpretive concerns, we described the excluded discharge survivors without MMSE data (*n* = 222) in Supplementary Table S2 and compared them with those with available MMSE (*n* = 414). The results showed that excluded patients tended to be older, more comorbid, and less likely to receive guideline-directed medical therapies (GDMT), reflecting a distinct frailty-enriched AHF readability risk phenotype. Although formal centralized inter-rater reliability auditing was not part of the original registry, the MMSE was administered face-to-face at discharge after clinical stabilization by certified cardiology residents under attending cardiologist supervision. All assessors received standardized cognitive-screening training, supervised real-patient practice, and periodic scoring feedback, and this process is now described in detail in the Methods as a real-world MMSE implementation benchmark.

Importantly, missing indicator Cox modeling and MMSE feasibility-weighted IPW analyses confirmed that the missing-MMSE indicator itself did not show an independent prognostic effect after multivariable- and spline-adjusted age modeling, while the composite GNRI–MMSE outcome ordering was preserved, supporting the practical prognostic weight of the composite screen rather than technical selection bias confounding the direction of the signal.

### 4.2. Relationship with Previous Literature and Biological Plausibility

Previous studies have established that malnutrition (low GNRI) and cognitive impairment (low MMSE) each predict adverse outcomes in HF independently. For example, Kinugasa et al. reported that GNRI < 92 predicted higher mortality after adjustment for conventional risk factors [15], while Dodson et al. and O’Donnell et al. showed that MMSE ≤ 23 was associated with increased rehospitalization and death [19,22]. In addition, multiple recent cohort studies have reinforced the prognostic utility of malnutrition indices in HF, including GNRI, CONUT, and PNI, demonstrating strong associations with all-cause mortality, HF readmission, and reduced exercise tolerance across both HFrEF and HFpEF populations [36,37]. Meta-analytic data further indicate that each 1-point decrease in GNRI is associated with a clinically meaningful increase in mortality risk [28]. However, few studies have simultaneously evaluated both domains. Our results extend this literature by demonstrating that the combination of low GNRI and low MMSE confers an additive prognostic burden beyond either factor alone, emphasizing the shared pathophysiological mechanisms linking nutritional depletion, neurocognitive dysfunction, and HF progression.

Potential biological pathways include systemic inflammation, neurohormonal activation, endothelial dysfunction, and impaired cerebral perfusion, which may contribute both to skeletal muscle wasting and cognitive decline [26]. Malnutrition further exacerbates protein–energy wasting, reduces albumin-mediated drug binding, and worsens catabolic signaling [38,39,40,41], whereas cognitive dysfunction impairs dietary intake, medication adherence, and timely care-seeking—together forming a self-reinforcing cycle of HF deterioration [42,43,44,45].

### 4.3. Clinical Implications and Translational Perspective

From a clinical standpoint, our findings highlight the importance of integrating routine GNRI and MMSE assessments into discharge evaluations for elderly patients with ADHF. A combined screening can be performed rapidly at the bedside using objective parameters (Alb, BW, simple cognitive tasks) and could help identify patients who would benefit from targeted post-discharge interventions—such as nutritional counseling, multidisciplinary rehabilitation, or cognitive support programs. Given the high prevalence of both conditions, we propose incorporating GNRI–MMSE evaluation in all patients aged ≥65 years before discharge, as part of a broader frailty assessment and transition-of-care planning. This approach could enhance individualized treatment, improve adherence, and ultimately reduce rehospitalization.

Importantly, incorporating this combined GNRI and MMSE stratification into clinical workflows may provide practical guidance at the time of discharge. For example, patients identified as high-risk (low GNRI/low MMSE) could be targeted for an enhanced multidisciplinary heart failure management program by providing a “vulnerable patient package” at discharge, including enhanced medication counseling by a pharmacist, high-protein dietary advice by a registered dietitian, and early outpatient follow-up within two weeks of discharge. Conversely, intermediate risk groups—such as those with isolated malnutrition or isolated cognitive impairment—may benefit from tailored interventions focused on the specific domain affected. Thus, this combined assessment has the potential to transition from simple risk identification to immediate post-discharge management planning.

### 4.4. Future Directions

Previous studies have demonstrated the usefulness of several interventions in improving malnutrition and cognitive impairment in patients with HF. The EFFORT and PICNIC trials have shown that the use of individualized nutritional support to reach nutritional goals resulted in a significant improvement in mortality in the short- and long-term, as well as in other clinical outcomes [46,47]. Previous studies have shown that optimal medical therapy and tailored management plans for HF, exercise training programs, and cognitive behavioral therapy are effective in preventing or improving cognitive impairment in patients with HF [48,49,50]. Although evidence in patients with HF remains limited and further clinical trials are necessary, these interventions have the potential to improve both malnutrition and cognitive impairment.

Furthermore, although our findings were robust across multiple sensitivity analyses, external validation in independent HF cohorts will be essential to confirm generalizability across different ethnicities, healthcare systems, and HF phenotypes. Prospective studies that incorporate serial assessments of both nutritional and cognitive status—and evaluate how dynamic changes relate to prognosis—may also provide stronger evidence for the causal pathways suggested in our analysis. Such validation will be a critical step toward broader adoption of the GNRI–MMSE composite as a standardized risk-stratification tool in clinical practice.

### 4.5. Limitations

This study has several limitations. First, this was a single-center study with a moderate sample size, and external validation in multi-ethnic cohorts is required to confirm generalizability beyond Japanese populations. Second, approximately one-third of eligible patients were excluded due to missing MMSE data, introducing potential selection bias toward those who were more clinically stable or cooperative at discharge. Third, we evaluated the nutritional status and cognitive function only once before discharge, and no information was obtained regarding these changes. Fourth, inter-rater reliability and standardized training for MMSE administration were not formally assessed, which may have introduced measurement variability. Fifth, residual confounding cannot be excluded despite multivariable adjustment. Sixth, only the total MMSE score was available in our dataset, and domain-specific subscores were not collected. As a result, we could not determine whether impairments in particular cognitive domains were more prevalent or more strongly associated with clinical outcomes. Future studies incorporating comprehensive neurocognitive assessments will be essential to clarify the domain-specific pathways linking cognition and prognosis. Seventh, educational attainment, a major determinant of MMSE performance, was not systematically collected in our registry. We were therefore unable to adjust for its influence in our models, and this may have contributed to cognitive-status misclassification or residual educational bias. We now explicitly acknowledge this limitation and highlight the need for future studies to incorporate educational data or alternative cognitive batteries less dependent on schooling to improve precision. Eighth, GNRI was calculated at discharge after clinical optimization rather than under strictly verified euvolemic conditions. Because body weight and serum albumin are sensitive to hydration status, inflammation, and acute catabolism during AHF recovery, GNRI at discharge may partly reflect residual congestion, posing a risk of nutritional misclassification. Although objective markers of congestion (e.g., IVC diameter, BNP trends, diuretic response) were routinely reviewed before discharge, future studies including standardized volume assessment (e.g., bioimpedance or congestion scores) under confirmed euvolemia are needed to further refine discharge-time nutritional classification. Finally, although our study focused on elderly patients, malnutrition and cognitive impairment also occur in younger individuals. Additional research is needed to evaluate their prevalence and prognostic significance in younger populations and to determine the extent to which our findings can be generalized beyond older adults.

## 5. Conclusions

In conclusion, the combined assessment of GNRI and MMSE at hospital discharge identifies elderly patients with ADHF who are at markedly increased risk of death and HF readmission. Malnutrition and cognitive impairment appear to act synergistically through biological and behavioral mechanisms. Routine incorporation of this dual-domain assessment into discharge planning may facilitate early risk stratification and guide multidisciplinary interventions aimed at improving long-term outcomes in this growing population.

## Figures and Tables

**Table 1:** Baseline characteristics.

	High-GNRI High-MMSE (N = 167)	Low-GNRI High-MMSE (N = 147)	High-GNRI Low-MMSE (N = 45)	Low-GNRI Low-MMSE (N = 55)	*p*-Value
Age, years	76 (71–80)	79 (74–84)	82 (77–86)	84 (81–88)	<0.001 b,c,e <0.05 a
Male, %	107 (64.1)	82 (55.8)	22 (48.9)	27 (49.1)	0.108
BMI, kg/m^2^	22.9 (21.4–25.1)	18.8 (16.9–20.5)	23.3 (21.0–25.8)	18.6 (15.9–20.3)	<0.001 a,c,d,f
SBP, mmHg	112 (101–126)	110 (98–122)	110 (98–122)	108 (99–122)	0.391
DBP, mmHg	62 (56–68)	60 (53–64)	62 (56–66)	60 (52–64)	<0.05 a
HR, beats/min	70 (60–76)	72 (63–82)	70 (62–77)	68 (61–79)	0.122
NYHA at discharge, %					<0.05 a,b
1	83 (49.7)	48 (32.7)	16 (35.6)	24 (43.6)	
2	82 (49.1)	89 (60.5)	24 (53.3)	26 (47.3)	
3	2 (1.2)	10 (6.8)	5 (11.1)	5 (9.1)	
4	0 (0)	0 (0)	0 (0)	0 (0)	
Medical history, %					
Hypertension	133 (79.6)	100 (68.0)	36 (80.0)	40 (72.7)	0.094
Dyslipidemia	89 (53.3)	63 (42.9)	20 (44.4)	16 (29.1)	<0.05 c
Diabetes mellitus	65 (38.9)	65 (44.2)	23 (51.1)	18 (32.7)	0.225
Cerebrovascular disease	29 (17.4)	17 (11.6)	12 (26.7)	12 (21.8)	0.072
CKD	126 (75.4)	112 (76.2)	40 (88.9)	40 (80.0)	0.250
COPD	19 (11.4)	16 (10.9)	4 (8.9)	7 (12.7)	0.942
Current or ex-smoker	105 (62.9)	87 (59.2)	24 (53.3)	24 (43.6)	0.080
Atrial fibrillation	70 (41.9)	55 (37.4)	15 (33.3)	27 (49.1)	0.339
Myocardial infarction	36 (21.6)	24 (16.3)	11 (24.4)	11 (20.0)	0.561
Medication at discharge, %					
ACE-I or ARB or ARNI	149 (89.2)	124 (84.4)	39 (86.7)	46 (83.6)	0.568
Beta-blockers	123 (73.7)	103 (70.1)	31 (68.9)	42 (76.4)	0.746
Aldosterone antagonists	81 (48.5)	62 (42.2)	21 (46.7)	29 (52.7)	0.526
SGLT2 inhibitor	32 (19.2)	34 (23.1)	11 (24.4)	8 (14.5)	0.488
Statin	93 (55.7)	68 (46.3)	22 (48.9)	15 (27.3)	<0.05 c
Diuretic	144 (86.2)	120 (81.6)	37 (82.2)	44 (80.0)	0.616
Loop diuretic	142 (85.0)	118 (80.3)	36 (80.0)	44 (80.0)	0.657
Loop diuretic dose, mg	28.0 ± 34.0	25.2 ± 16.6	27.3 ± 15.7	26.8 ± 17.9	0.817
Tolvaptan	34 (20.4)	36 (24.5)	11 (24.4)	12 (21.8))	0.829
Laboratory data					
Hb, g/dL	12.1 (10.7–13.3)	10.6 (9.6–11.9)	11.6 (10.2–12.6)	10.4 (9.8–11.7)	<0.001 a,c <0.05 d
Alb, g/dL	3.9 (3.7–4.0)	3.4 (3.1–3.6)	3.7 (3.6–4.0)	3.3 (3.1–3.6)	<0.001 a,c,d,f
BUN, mg/dL	23.0 (18.0–32.0)	24.0 (17.0–36.5)	30.0 (21.0–40.0)	36.0 (21.5–46.5)	<0.05 c,e
Cr, mg/dL	1.18 (0.93–1.55)	1.09 (0.81–1.88)	1.33 (0.91–1.75)	1.41 (0.99–1.69)	0.333
eGFR, mL/min/1.73 m^2^	34.3 (25.1–46.0)	37.6 (20.4–51.6)	29.2 (22.3–45.2)	27.2 (22.6–41.2)	0.235
Uric acid	6.8 (5.7–8.5)	6.3 (4.7–7.3)	7.1 (5.2–8.1)	7.2 (5.8–9.0)	<0.05 a,e
Serum sodium, mEq/L	140 (139–142)	139 (137–141)	138 (135–142)	138 (135–140)	<0.001 c <0.05 a
Serum potassium, mEq/L	4.2 (3.8–4.5)	4.1 (3.8–4.4)	4.3 (4.1–4.5)	4.0 (3.8–4.6)	0.146
BNP, pg/mL	334 (178–502)	352 (227–756)	278 (155–497)	357 (169–493)	0.119
LVEF, %	45.5 (34.8–61.3)	44.0 (37.3–60.0)	44.0 (35.0–61.0)	49.0 (37.0–63.0)	0.867
Heart failure phenotypes					0.607
HFrEF	65 (38.9)	47 (32.0)	16 (35.6)	19 (34.5)	
HFmrEF	34 (20.4)	41 (27.9)	12 (26.7)	10 (18.2)	
HFpEF	68 (40.7)	59 (40.1)	17 (37.8)	26 (47.3)	

*p*-value refers to comparisons of the means, median, or proportions among the groups by the one-way ANOVA, Kruskal–Wallis, and Pearson Chi-square tests with Bonferroni post hoc analysis. ACE-I, angiotensin-converting enzyme inhibitor; Alb, albumin; ARB, angiotensin II receptor blocker; ARNI, angiotensin receptor neprilysin inhibitor; BMI, body mass index; BNP, brain natriuretic peptide; BUN, blood urea nitrogen; CKD, chronic kidney disease; COPD, chronic obstructive pulmonary disease; Cr, creatinine; DBP, diastolic blood pressure; eGFR, estimated glomerular filtration rate; GNRI, geriatric nutritional risk index; Hb, hemoglobin; HFmrEF, heart failure with mid-range ejection fraction; HFpEF, heart failure with preserved ejection fraction; HFrEF, heart failure with reduced ejection fraction; HR, heart rate; LVEF, left ventricular ejection fraction; MMSE, mini-mental state examination; NYHA, New York Heart Association; SBP, systolic blood pressure.Values are n (%) or median [interquartile range]. Body mass index is the weight in kilograms divided by the square of the height in meters. ^a^ high-GNRI/high-MMSE score versus low GNRI/high MMSE score. ^b^ high-GNRI/high-MMSE score versus high GNRI/low MMSE score. ^c^ high-GNRI/high-MMSE score versus low GNRI/low MMSE score. ^d^ low-GNRI/high-MMSE score versus high GNRI/low MMSE score. ^e^ low-GNRI/high-MMSE score versus low-GNRI/low-MMSE score. ^f^ high-GNRI/low-MMSE score versus low-GNRI/low-MMSE score.

**Table 2:** Cox regression analysis for the combined endpoint and all-cause death in ADHF patients.

	Univariate Analysis		Multivariate Analysis	
	HR (95%CI)	*p*-Value	HR (95%CI)	*p*-Value
All-cause death or HF readmission				
high-GNRI high-MMSE	1 (reference)		1 (reference)	
low-GNRI high-MMSE	1.689 (1.220–2.338)	0.002	1.556 (1.031–2.349)	0.035
high-GNRI low-MMSE	1.743 (1.114–2.729)	0.015	1.458 (0.843–2.524)	0.177
low-GNRI low-MMSE	3.127 (2.129–4.592)	<0.001	2.163 (1.284–3.643)	0.004
All-cause death				
high-GNRI high-MMSE	1 (reference)		1 (reference)	
low-GNRI high-MMSE	1.997 (1.373–2.904)	<0.001	1.568 (0.996–2.468)	0.052
high-GNRI low-MMSE	1.613 (0.937–2.778)	0.085	1.210 (0.644–2.276)	0.554
low-GNRI low-MMSE	3.681 (2.387–5.675)	<0.001	2.209 (1.222–3.993)	0.009

## Data Availability

The data that support the findings of this study are available from the corresponding author upon reasonable request.

## Supplementary Materials

The following supporting information can be downloaded at: https://www.mdpi.com/article/10.3390/nu18020189/s1, Table S1: Definitions of GNRI and MMSE; Table S2: Baseline characteristics (MMSE vs. no-MMSE); Table S3: Cox regression analysis for the combined endpoint and all-cause death in ADHF patients with Age ≥ 80 years and Age < 80 years; Table S4: Cox regression analysis for the combined endpoint and all-cause death in ADHF patients with ejection fraction ≤ 40% (HFrEF) and ejection fraction > 40% (non HFrEF); Figure S1. Prognostic association of discharge GNRI and MMSE modeled as continuous standardized variables with age-adjusted restricted cubic splines; Figure S2. Kaplan–Meier analyses in the combined assessment of the GNRI and MMSE at discharge after excluding patients with extreme GNRI values (<82) for postdischarge all-cause death and heart failure rehospitalization, and all-cause death.

## Abbreviations

The following abbreviations are used in this manuscript:

ACE-I	angiotensin-converting enzyme inhibitor
ADHF	acute decompensated heart failure
Alb	albumin
ARB	angiotensin II receptor blocker
ARNI	angiotensin receptor neprilysin inhibitor
BNP	B-type natriuretic peptide
BUN	blood urea nitrogen
eGFR	estimated glomerular filtration rate
GNRI	geriatric nutritional risk index
Hb	hemoglobin
HF	heart failure
HR	hazard ratios
LVEF	left ventricular ejection fraction
MMSE	mini-mental state examination
NARA-LOGEVITY	Nara Longevity Registry and Analyses
SGLT2	sodium-glucose cotransporter 2
