# Supplementary material for: Prognostic Impact of Combined Nutritional and Cognitive Status on Long-Term Outcome in Acute Decompensated Heart Failure
Source: Nutrients. 2026 Jan 6;18(2):189. doi: 10.3390/nu18020189 (PMC12844809; doi:10.3390/nu18020189)
Supplement: Supplementary file 1 [file nutrients-18-00189-s001.zip › nutrients-3997905-supplementary.pdf]

Supplementary Material

Supplementary Table S1. Definitions of GNRI and MMSE

Parameter	Definition / Description
Geriatric Nutritional Risk Index (GNRI)	The Geriatric Nutritional Risk Index (GNRI) was calculated using the formula:<br>GNRI = 14.89 × Albumin (g/dL) + 41.7 × (body weight / ideal body weight)<br><br>Ideal body weight was calculated as follows [1]:<br>• Men: height (cm) − 100 − [(height (cm) − 150) / 4]<br>• Women: height (cm) − 100 − [(height (cm) − 150) / 2.5]<br><br>According to Bouillanne et al. [1], GNRI cut-off values indicate four grades of nutritional risk:<br>• Major risk: GNRI < 82<br>• Moderate risk: 82 ≤ GNRI < 92<br>• Low risk: 92 ≤ GNRI ≤ 98<br>• No risk: GNRI > 98<br><br>In this study, patients were categorized into:<br>• Low-GNRI group (GNRI < 92)<br>• High-GNRI group (GNRI ≥ 92).
Mini-Mental State Examination	The Mini-Mental State Examination (MMSE) was used to assess cognitive function at discharge. It includes 10 domains: orientation to time (5 points), orientation to place (5 points), registration (3

(MMSE)	points), attention and calculation (5 points), recall (3 points), naming and repetition (3 points), comprehension (3 points), reading ability (1 point), writing ability (1 point), and design copy (1 point). The total score ranges from 0 to 30, with higher scores indicating better cognitive performance.<br><br>Patients were classified into:<br>• Low-MMSE group (MMSE ≤ 23): at risk of cognitive impairment.<br>• High-MMSE group (MMSE > 23): low or no risk of cognitive impairment.<br><br>A score of 23 was used as the conventional cut-off for cognitive impairment [2].

GNRI, geriatric nutritional risk index; MMSE, mini-mental state examination.

Supplementary Table S2. Baseline characteristics (MMSE vs. no-MMSE)

	MMSE (N=414)	No-MMSE (N=222)	P-value
Age, years	79 (73-84)	81 (75-87)	<0.001
Male, %	238 (57.5)	115 (51.8)	0.196
BMI, kg/m^2	20.9 (18.5-23.5)	20.0 (17.8-23.3)	0.171
SBP, mmHg	110 (100-124)	112 (100-124)	0.444
DBP, mmHg	60 (54-66)	60 (54-67)	0.645
HR, beats/min	70 (62-78)	70 (65-82)	0.011
NYHA at discharge, %			<0.001

1	171 (41.3)	47 (21.2)	
2	221 (53.4)	138 (62.2)	
3	22 (5.3)	29 (13.1)	
4	0 (0)	8 (3.6)	
Medical history, %			
Hypertension	309 (74.6)	173 (77.9)	0.409
Dyslipidemia	188 (45.4)	91 (41.0)	0.324
Diabetes mellitus	177 (42.8)	93 (41.9)	0.832
Cerebrovascular disease	70 (16.9)	36 (16.2)	0.911
CKD	322 (77.8)	174 (78.4)	0.941
COPD	46 (11.1)	24 (10.8)	0.999
Current or ex-smoker	240 (58.0)	109 (49.1)	0.039
Atrial fibrillation	167 (40.3)	89 (40.1)	0.999
Myocardial infarction	82 (19.8)	46 (20.7)	0.865
Medication at discharge, %			
ACE-I or ARB or ARNI	358 (86.5)	178 (80.2)	0.050
Beta-blockers	299 (72.2)	139 (62.6)	0.016
Aldosterone antagonists	193 (46.6)	88 (39.6)	0.108
SGLT2 inhibitor	85 (20.5)	46 (20.7)	0.999
Statin	198 (47.8)	83 (37.4)	0.015
Diuretic	345 (83.3)	164 (73.9)	0.006
Loop diuretic	340 (82.1)	157 (70.7)	0.001
Loop diuretic dose, mg	28.0±34.0	24.6±19.3	0.154

Tolvaptan	93 (22.5)	49 (22.1)	0.989
Laboratory data			
Hb, g/dL	11.2 (10.1-12.6)	10.7 (9.6-12.0)	0.001
Alb, g/dL	3.6 (3.3-3.9)	3.4 (3.0-3.6)	<0.001
BUN, mg/dL	25.0 (18.0-37.0)	27.0 (19.0-38.0)	0.189
Cr, mg/dL	1.19 (0.87-1.65)	1.15 (0.87-1.79)	0.988
eGFR, mL/min/1.73 m^2	33.7 (23.5-47.4)	35.2 (21.6-48.1)	0.955
Uric acid	6.7 (5.3-8.1)	6.9 (5.3-8.3)	0.545
Serum sodium, mEq/L	139 (137-141)	139 (136-141)	0.173
Serum potassium, mEq/L	4.2 (3.8-4.6)	4.1 (3.7-4.6)	0.326
BNP, pg/mL	338 (188-567)	366 (182-777)	0.255
LVEF, %	45.0 (35.0-61.0)	49.0 (37.0-62.0)	0.126
Heart failure phenotypes			0.439
HFrEF	147 (35.5)	72 (34.1)	
HFmrEF	97 (23.4)	42 (19.9)	
HFpEF	170 (41.1)	97 (46.0)	

ACE-I, angiotensin-converting enzyme inhibitor; Alb, albumin; ARB, angiotensin II receptor blocker; ARNI, angiotensin receptor neprilysin inhibitor; BMI, body mass index; BNP, brain natriuretic peptide; BUN, blood urea nitrogen; CKD, chronic kidney disease; COPD, chronic obstructive pulmonary disease; Cr, creatinine; DBP, diastolic blood pressure; eGFR, estimated glomerular filtration rate; GNRI, geriatric nutritional risk index; Hb, hemoglobin; HFmrEF, heart failure with mid-range ejection fraction; HFpEF, heart failure with

preserved ejection fraction; HFrEF, heart failure with reduced ejection fraction; HR, heart rate; LVEF, left ventricular ejection fraction;

MMSE, mini-mental state examination; NYHA, New York Heart Association; SBP, systolic blood pressure.

Values are n (%) or median [interquartile range].

Body mass index is the weight in kilograms divided by the square of the height in meters.

Supplementary Table S3. Cox regression analysis for the combined endpoint and all-cause death in ADHF patients with Age≥80 years and Age<80 years

	Age≥80 years		Age<80 years	
	HR (95%CI)	P-value	HR (95%CI)	P-value
All-cause death or HF readmission				
high-GNRI high-MMSE	1 (reference)		1 (reference)	
low-GNRI high-MMSE	1.423 (0.888-2.282)	0.143	1.559 (0.980-2.480)	0.061
high-GNRI low-MMSE	1.368 (0.753-2.484)	0.304	1.611 (0.780-3.327)	0.197
low-GNRI low-MMSE	2.474 (1.513-4.046)	<0.001	1.493 (0.533-4.180)	0.445

P for interaction		0.707		
All-cause death				
high-GNRI high-MMSE	1 (reference)		1 (reference)	
low-GNRI high-MMSE	1.845 (1.085-3.139)	0.024	1.603 (0.919-2.798)	0.097
high-GNRI low-MMSE	1.281 (0.637-2.578)	0.487	1.337 (0.515-3.472)	0.551
low-GNRI low-MMSE	2.992 (1.722-5.199)	<0.001	1.063 (0.253-4.470)	0.934
P for interaction		0.381		

GNRI, geriatric nutritional risk index; MMSE, mini-mental state examination.

Supplementary Table S4. Cox regression analysis for the combined endpoint and all-cause death in ADHF patients with ejection fraction≤40% (HFrEF) and ejection fraction>40% (non HFrEF)

	HFrEF		non HFrEF	
	HR (95%CI)	P-value	HR (95%CI)	P-value
All-cause death or HF readmission				
high-GNRI high-MMSE	1 (reference)		1 (reference)	

low-GNRI high-MMSE	1.496 (0.821-2.725)	0.188	1.739 (1.176-2.572)	0.006
high-GNRI low-MMSE	1.568 (0.701-3.507)	0.273	1.800 (1.048-3.091)	0.033
low-GNRI low-MMSE	3.559 (1.844-6.871)	<0.001	2.880 (1.793-4.627)	<0.001
P for interaction		0.546		
All-cause death				
high-GNRI high-MMSE	1 (reference)		1 (reference)	
low-GNRI high-MMSE	1.519 (0.834-2.766)	0.172	1.998 (1.280-3.119)	0.002
high-GNRI low-MMSE	1.732 (0.774-3.873)	0.181	1.448 (0.745-2.814)	0.275
low-GNRI low-MMSE	3.378 (1.756-6.499)	<0.001	3.575 (2.113-6.049)	<0.001
P for interaction		0.668		

GNRI, geriatric nutritional risk index; MMSE, mini-mental state examination.

Supplementary Figure S1. Prognostic association of discharge GNRI and MMSE modeled as continuous standardized variables with age-adjusted restricted cubic splines

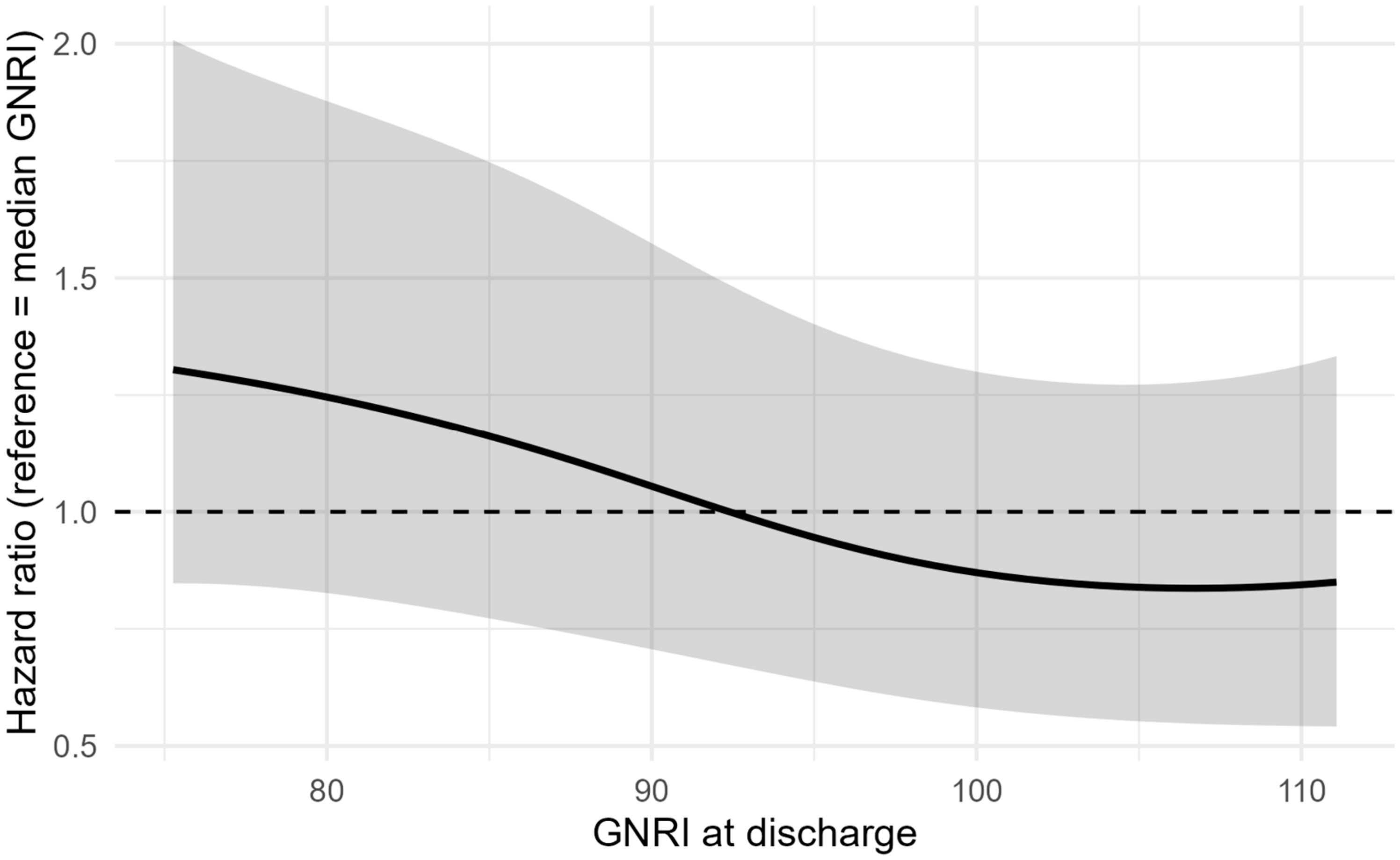

(A)

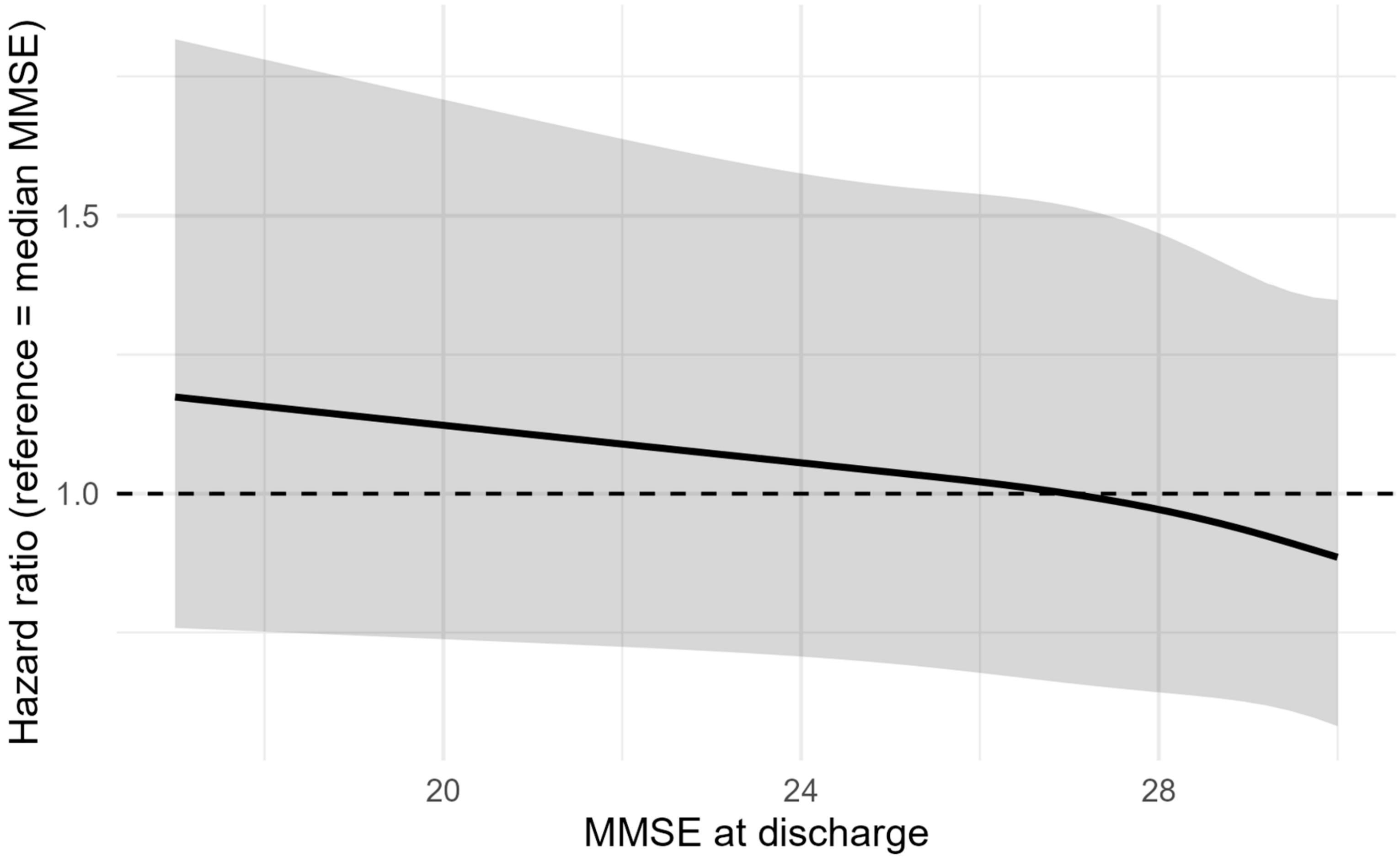
Hazard ratio (reference = median MMSE)
1.5
1.0
20
24
28
MMSE at discharge

(B)

Supplementary Figure S2. Kaplan–Meier analyses in the combined assessment of the GNRI and MMSE at discharge after excluding patients with extreme GNRI values (<82) for postdischarge all-cause death and heart failure rehospitalization, and all-cause death.

All-cause death and Heart failure rehospitalization

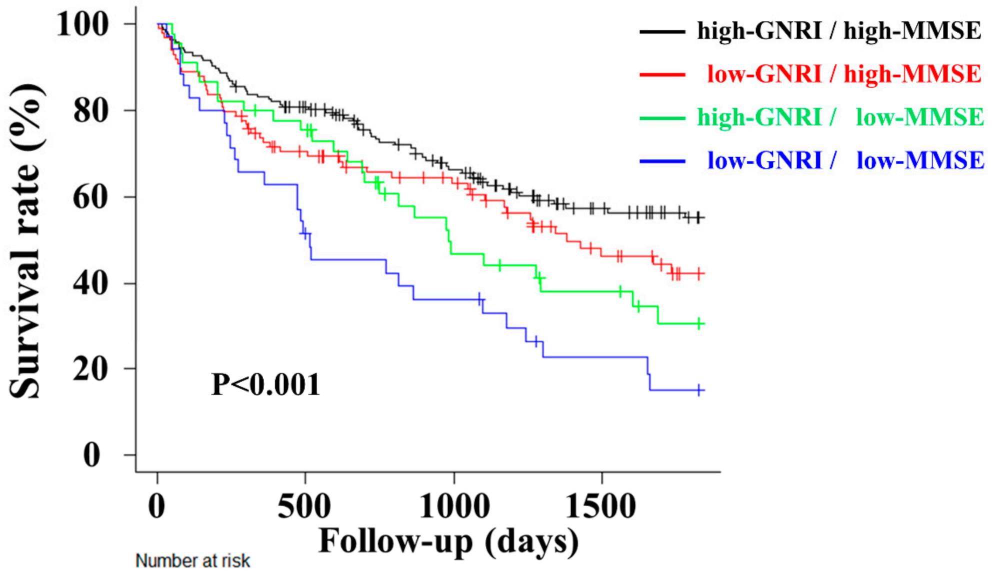

Number at risk

	0	500	1000	1500
high-GNRI / high-MMSE	167	129	91	58
low-GNRI / high-MMSE	99	64	48	26
high-GNRI / low-MMSE	45	33	17	12
low-GNRI / low-MMSE	35	17	12	6

high-GNRI/high-MMSE vs. low-GNRI/low-MMSE $p<0.001$

low-GNRI/high-MMSE vs. low-GNRI/low-MMSE $p=0.022$

(A)

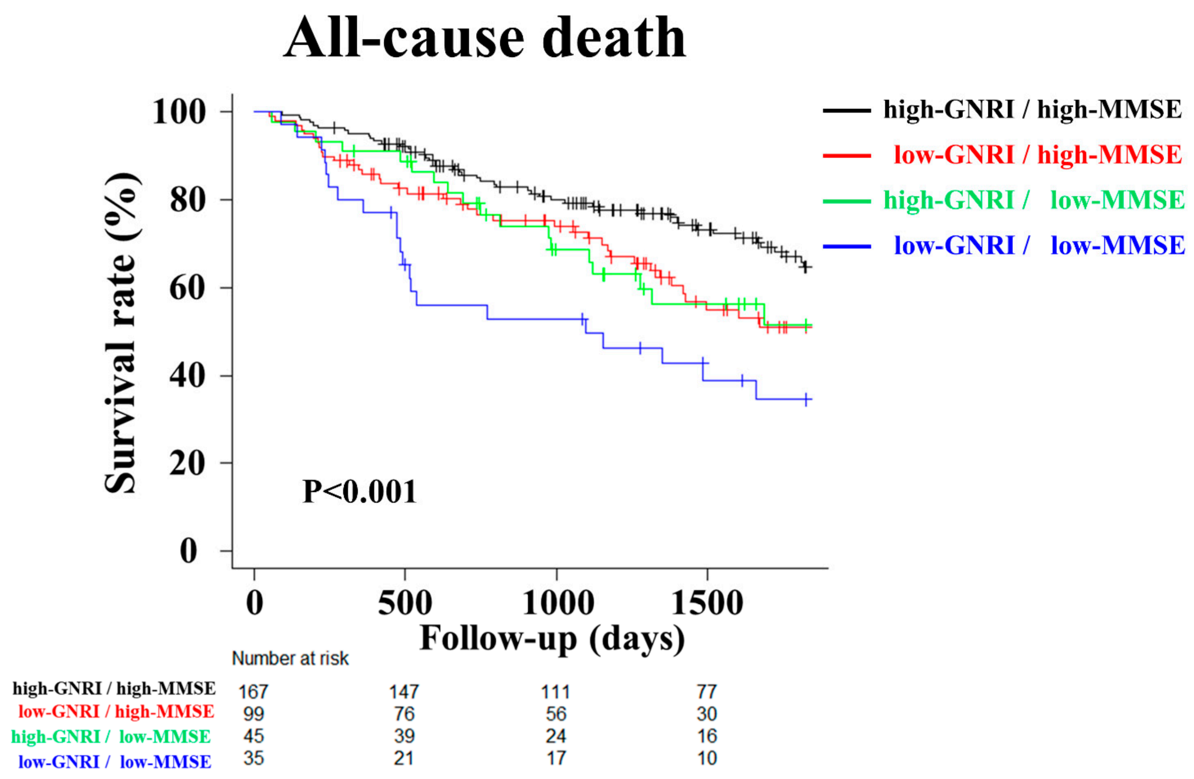

high-GNRI/high-MMSE vs. low-GNRI/low-MMSE p=0.001

(B)

Figure legends:

Supplementary Figure S1A:

Restricted cubic spline (RCS) analysis for discharge GNRI and risk of the composite endpoint (all-cause death or heart failure readmission). The model adjusted for age, sex, NYHA class at discharge, diabetes mellitus, Hb, BNP, LVEF, BUN, creatinine, serum sodium, systolic blood pressure at discharge, and the use of ACE-I/ARB/ARNI, Beta-blockers, Aldosterone antagonists, and SGLT2 inhibitor. GNRI was entered as a standardized variable, and the spline curve shows an approximately linear inverse association across the central GNRI range, with confidence intervals widening at extreme GNRI values.

Supplementary Figure S1B:

RCS analysis for discharge MMSE score and composite endpoint risk. The model included the same multivariable adjustment set as in 1A. MMSE was analyzed as a standardized continuous variable. The spline curve demonstrates a generally inverse, near-linear trend between higher MMSE and lower risk within the central score range, although the relative effect estimate did not reach conventional statistical significance. Uncertainty increased at score extremes due to sparse data density.

Supplementary Figure S2:

Kaplan–Meier curve analyses showed that the low-GNRI/low-MMSE group was associated with a higher event rate than the high-GNRI/high-MMSE group for both the combined endpoint (2A) and all-cause death (2B).

References

1. Bouillanne, O.; Morineau, G.; Dupont, C.; Coulombel, I.; Vincent, J.P.; Nicolis, I.; Benazeth, S.; Cynober, L.; Aussel, C. Geriatric Nutritional Risk Index: a new index for evaluating at-risk elderly medical patients. *Am J Clin Nutr* **2005**, *82*, 777-783, doi:10.1093/ajcn/82.4.777.

2. Anthony, J.C.; LeResche, L.; Niaz, U.; von Korff, M.R.; Folstein, M.F. Limits of the 'Mini-Mental State' as a screening test for dementia and delirium among hospital patients. *Psychol Med* **1982**, *12*, 397-408, doi:10.1017/s0033291700046730.
